# Editorial: Molecular diagnostics for infectious diseases: Novel approaches, clinical applications and future challenges

**DOI:** 10.3389/fmicb.2023.1153827

**Published:** 2023-03-03

**Authors:** Bing Gu, Chao Zhuo, Xiaogang Xu, Kamal El Bissati

**Affiliations:** ^1^Division of Laboratory Medicine, Guangdong Provincial People's Hospital, Guangzhou, China; ^2^Guangzhou Institute of Respiratory Health, First Affiliated Hospital of Guangzhou Medical University, Guangzhou, China; ^3^Institute of Antibiotics, Huashan Hospital, Fudan University, Shanghai, China; ^4^Institute for Molecular Engineering, University of Chicago Medical Center, Chicago, IL, United States

**Keywords:** infectious diseases, molecular diagnostics, mNGS, CRISPR-Cas based assay, POCT

In this Research Topic of Frontiers in Microbiology, we assembled a collection of 12 original research articles and 2 reviews within the theme “*Molecular Diagnostics for Infectious Diseases: Novel approaches, Clinical Applications and Future Challenges*.” The intended goal of this Research Topic was to present an updated view of current innovations in molecular diagnostics and challenges against clinical pathogens. The incidence of infectious diseases is high and often causes critical illness, which seriously threatens human life and health. The diagnosis and treatment depend on etiological detection techniques. Therefore, etiological detection technology is required to achieve timely, accurate, and comprehensive detection of pathogens. The traditional etiological diagnosis methods of infection mainly include morphological identification, microbial culture, smear microscopic examination, antigen and antibody detection, nucleic acid detection, etc. However, these methods have many limitations, such as long detection cycles, low sensitivity, and narrow detection spectrum of pathogens, especially for infections caused by rare or new pathogens. Traditional detection methods cannot effectively address them. Therefore, there is an urgent need for a series of more powerful pathogen detection tools. Metagenomic next-generation sequencing (mNGS) is a powerful pathogen detection approach that can potentially diagnose nucleic acids (DNA and RNA) of all infectious diseases, including bacteria, fungi, viruses, and parasites, in a single test. It has been recognized as a subversive technique for pathogen detection, especially for rare and complex infectious diseases. Specific High Sensitivity Enzymatic Reporter UnLOCKing (SHERLOCK) is a platform molecular diagnostic based on CRISPR-Cas enzymology for specific recognition of desired DNA or RNA sequences. It has been used to detect viruses such as Zika, dengue, and African classical swine fever virus. Although mNGS and SHERLOCK have played an active role in diagnosing clinical infection, they still face many limitations and challenges in clinical applications (e.g., quality control and standardization, distinguishing pathogenic pathogens from colonized pathogens, stability, simplicity, and diversity of targets). In light of these challenges, new research strategies have appeared that enhance the possibility of traditional pathogen detection methods. This Research Topic evaluates these new possibilities and brings together authorities in this field and experiences that will focus on the clinical applications, advantages, and challenges of all aspects of molecular approaches for pathogen diagnostics. This Research Topic includes but is not limited to (i) progression of methodology for pathogen diagnostics, (ii) sequencing-based applications in diagnostics of clinical infection, (iii) progression of databases and analysis procedures for clinical application of metagenomics, (iv) CRISPR-Cas based pathogen identification, and (v) discovery of candidate biomarkers for infection diagnostics.

The paper of Lin Z. et al. reported the development of the visual method, dRAA-CRISPR/Cas12a, in detecting the expression of the virulent genes *aerA* and/or *hlyA* of *Aeromonas hydrophila*. *A. hydrophila* is a re-emerging aquaculture and waterborne/foodborne pathogen to humans. This method is rapid and highly sensitive. The results of the expression of the genes can be read using a UV flashlight. The authors showed the prospect of this promising method in the early diagnosis of *A. hydrophila* infection and on-site detection, especially in resource-poor areas of this pathogen in food and aquaculture. In another earlier study, Wang et al. used a similar technique to accurately identify and detect *Staphylococcus aureus* in clinical samples. Ummarino et al. describe a novel PCR-based method for the diagnosis of *Enterobius vermicularis* in stool samples, specifically designed for clinical application. This is the first time that such a technique has been used to detect these nematodes in fecal samples. The advantages of this method can help in the identification and molecular characterization of parasite species. However, parasitological methods and coprological analysis (especially eggs) in feces remain the gold standard for this detection.

In addition, Chen W. et al. reviewed current knowledge about the current molecular diagnostic methods of swine vesicular diseases, such as Foot-and-Mouth Disease Virus (FMDV), SenecaVirus A (SVA), and Swine Vesicular Disease Virus (SVDV). All these viruses are members of the family Picornaviridae, which can cause vesicular lesions in the tissues of the mouth, nose, feet, skin, and mucous membrane of animals. RT-PCR and real-time RT-PCR are still the most reliable and gold standard methods for detecting viruses, including FMDV, SVA, and SVDV. The authors illustrate novel molecular diagnostics of vesicular diseases. These include loop-mediated isothermal amplification (LAMP), recombinase polymerase amplification (RPA), Luminex, and CRISPR-Cas technology. Luminex technology has been developed for the differential diagnosis of FMDV, SVDV, and other vesicular disease pathogens. In addition, the PCR-based fluorescent Luminex assay was suitable for human papillomavirus (HPV) genotyping. The assay may be used to provide critical clinical information for the early detection of HPV.

Havasi et al. reviewed the similarities and differences in the diagnosis of influenza A, influenza B, and SARS-CoV-2. They discussed the clinical presentation of influenza and SARS-CoV-2 and techniques available for diagnosis. Furthermore, they summarized available data regarding the multiplex diagnostic assay of both viral infections. Lin Y. et al. developed a rapid PCR-based nanopore adaptive sequencing method (RPNAS) to enrich the viral genome directly from respiratory samples. The same method was further applied successfully for the enrichment of SARS-CoV-2. These findings promise to improve viral clinical detection and genome surveillance sensitivity and timeliness.

Porcine circovirus (PCV) type 2 has been identified as a causative agent of post-weaning multisystem wasting syndrome (PMWS), an economically important multifactorial disease in the swine industry worldwide. Li et al. developed a dual nested PCR detection method to monitor PCV types 2 and 3 simultaneously. This method provides an effective tool for molecular epidemiological studies and blood sample detection of PCV.

Zhang et al. chose to focus their work on the molecular characteristics, susceptibility, and prevalence of infection of *Cryptococcus* spp. to understand the epidemiology of this disease in a province of China. They generated a large amount of data from 180 strains of *Cryptococcus* using the WGS method. These data will lay a good foundation for the subsequent development of molecular detection methods for *Cryptococcus* spp.

*Chlamydia trachomatis* is one of the most common pathogens causing sexually transmitted infections. In their study, Zhao et al. used next-generation high-throughput sequencing (NGHTS) to determine *C. trachomatis* genotypes, particularly mixed-genotype infections, and their association with clinical manifestations. The results indicated that NGHTS is suitable for identifying *C. trachomatis* mixed-genotype infections. In another study, Chen X. et al. integrated nanoparticle-based lateral flow biosensors with LAMP for rapid and visual identification of *C. trachomatis* for point-of-care, which may provide a convenient testing tool for chlamydial infection screening.

*Haemophilus influenza*e is a common human pathogen that causes a range of infectious diseases in children and adults. Cheng et al. reported the clinical application of the LAMP method to rapidly detect *H. influenzae*. The assay provides rapid, accurate, and sensitive detection, making it a promising screening strategy in clinical laboratory settings. In another paper, Slotved et al. evaluated the use of whole genome sequencing (WGS) in national surveillance and characterization of *H. influenza* clinical isolates in Denmark. Their results show 100% concordance between phenotypic serotyping methods and WGS-based approaches.

Cutaneous leishmaniasis is a disease caused by protozoan parasites of the genus *Leishmania* and is a major public health problem in many parts of Latin America. Dueñas et al. developed CRISPR-based molecular tools to diagnose *Leishmania* infections at the genus and *L*. (*Viannia*) subgenus levels. Visceral leishmaniasis (Kala-Azar) is uncommon in China. Gao et al. reported two Kala-Azar cases in China diagnosed by mNGS. Their reports suggest that mNGS detection is beneficial for diagnosing and treating infectious diseases with unknown causes.

In conclusion, this collection includes several excellent studies summarizing the latest advances in pathogenic molecular detection technology and accumulating a large amount of pathogenic genomic data. This collection will promote the integration and utilization of molecular detection technology and pathogens genomic data in the future and establish simpler, faster, and more accurate infection pathogen detection methods. Creating a system that integrates pathogen genomic sequences from numerous ongoing surveillance efforts, such as foodborne and hospital-acquired, would allow for automated real-time analyses (Jackson et al., 2016; Stevens et al., 2022). This can rapidly identify cluster-related pathogen genome sequences, potential transmission chains, and alerts for disease outbreaks. Finally, developing new detection methods should consider the economic component of low and middle-income countries for universal equality and one health world.

## Author contributions

KE, XX, CZ, and BG drafted and edited the editorial. All authors contributed to the article and approved the submitted version.
